# The Impact of Ageing on 11C-Hydroxyephedrine Uptake in the Rat Heart

**DOI:** 10.1038/s41598-018-29509-0

**Published:** 2018-07-24

**Authors:** Rudolf A. Werner, Xinyu Chen, Yoshifumi Maya, Christoph Eissler, Mitsuru Hirano, Naoko Nose, Hiroshi Wakabayashi, Constantin Lapa, Mehrbod S. Javadi, Takahiro Higuchi

**Affiliations:** 1 Department of Nuclear Medicine, University of Würzburg, Würzburg, Germany; 2 The Russell H. Morgan Department of Radiology and Radiological Science, Division of Nuclear Medicine and Molecular Imaging, Johns Hopkins University School of Medicine, Baltimore, MD United States; 3 Comprehensive Heart Failure Center (CHFC), University of Würzburg, Würzburg, Germany; 4 Research Centre, Nihon Medi-Physics Co., Ltd., Chiba, Japan; 5 Department of Biomedical Imaging, National Cerebral and Cardiovascular Center, Suita, Japan

## Abstract

We aimed to explore the impact of ageing on 11C-hydroxyephedrine (11C-HED) uptake in the healthy rat heart in a longitudinal setting. To investigate a potential cold mass effect, the influence of specific activity on cardiac 11C-HED uptake was evaluated: 11C-HED was synthesized by N-methylation of (−)-metaraminol as the free base (radiochemical purity >95%) and a wide range of specific activities (0.2–141.9 GBq/μmol) were prepared. ^11^C-HED (48.7 ± 9.7MBq, ranged 0.2–60.4 μg/kg cold mass) was injected in healthy Wistar Rats. Dynamic 23-frame PET images were obtained over 30 min. Time activity curves were generated for the blood input function and myocardial tissue. Cardiac 11C-HED retention index (%/min) was calculated as myocardial tissue activity at 20–30 min divided by the integral of the blood activity curves. Additionally, the impact of ageing on myocardial 11C-HED uptake was investigated longitudinally by PET studies at different ages of healthy Wistar Rats. A dose-dependent reduction of cardiac 11C-HED uptake was observed: The estimated retention index as a marker of norepinephrine function decreased at a lower specific activity (higher amount of cold mass). This observed high affinity of 11C-HED to the neural norepinephrine transporter triggered a subsequent study: In a longitudinal setting, the 11C-HED retention index decreased with increasing age. An age-related decline of cardiac sympathetic innervation could be demonstrated. The herein observed cold mass effect might increase in succeeding scans and therefore, 11C-HED microPET studies should be planned with extreme caution if one single radiosynthesis is scheduled for multiple animals.

## Introduction

As the predominant disorder of the ageing population, heart failure (HF) is the major cause of death in both the United States and Europe^1,2^. In this regard, HF can be understood as the result of cardiovascular ageing, representing the convergence of age-related alterations in both cardiovascular structure and function^3^.

Increasing interest in the age-dependent alterations in myocardial sympathetic nerve integrity has been aroused in particular from recognition that neurohumoral mechanisms may be the cause for the age-related increase in cardiovascular morbidity and mortality^4–7^. Deterioration in cardiac innervation in the elderly population is characterized by an elevated plasma concentration of the neurotransmitter norepinephrine (NE)^8,9^, an increased firing rate in the postganglionic fibers to the skeletal muscle^10,11^, impaired function of the NE transporter (uptake-1 mechanism)^4,5^ and reduced plasma clearance of NE in the synaptic cleft^12,13^.

The diagnostic use of cardiac radionuclide imaging probes such as 123I-metaiodobenzylguanidine (123I-mIBG) for Single Photon Emission Computed Tomography (SPECT) or 11C-hydroxyephedrine (11C-HED) for Positron Emission Tomography (PET) is currently expanding^14–17^. Both radiotracers are considered to reflect sympathetic presynaptic function, as they share NE pathways and therefore interact with uptake-1 mechanism, which recovers exocitotically released NE from the synaptic cleft^18–21^. An extensive body of evidence has been reported on the utility for risk stratification among severe HF patients using both imaging agents^22–24^: In the prospective Prediction of ARrhythmic Events with Positron Emission Tomography (PAREPET) trial, sympathetic neuronal impairment assessed by 11C-HED predicted sudden cardiac arrest independently of left ventricular ejection fraction^25^. Apart from that, the impact of ageing on cardiac innervation assessed by 123I-mIBG has also been reported previously, e.g. in healthy subjects^26–29^ or in patients suffering from systolic HF^30^. Of note, Rengo *et al*. even suggested an age-dependent adjustment of the well-established 123I-mIBG heart-to-mediastinum ratios, which are frequently used for stratifying the risk of cardiac events^30^.

Hence, given the expected broadened use of 11C-HED outside of controlled clinical trials as well as the alterations of myocardial sympathetic nerve function in the elderly, we aimed to explore the potential impact of ageing on cardiac 11C-HED uptake in healthy rats in a longitudinal setting.

## Materials and Methods

Animal protocols were approved by the local Animal Care and Use Committee (National Cardiovascular and Cerebral Research Center, Suita, Japan) and conducted according to the Guide for the Care and Use of Laboratory Animals (NIH Publication No. 85-23, revised 1996)^31^.

### Study Design

The first study was performed to explore a potential cold mass effect on myocardial 11C-HED uptake. Thereafter, the impact of ageing on cardiac ^11^C-HED uptake was examined in a longitudinal setting.

### Imaging Protocols

All animals were maintained under anesthesia throughout the imaging procedure with 2% isoflurane. 11C-HED imaging was performed using a micro PET system (Inveon; Siemens Healthcare, Erlangen, Germany). Its characteristics have been described in^32^. Prior to a bolus tracer injection of 11C-HED (50 MBq) via the tail vein, a list mode 30 min image acquisition was started. The list mode data was reconstructed into a dynamic sequence (23 frames: 15 × 8 s, 3 × 60 s, 5 × 300 s) using ordered-subset expectation maximization with 16 subsets and 4 iterations^33^. For the second study (impact of ageing on myocardial 11C-HED uptake), a reference scan with 18F-fluorodeoxyglucose (18F-FDG) was performed after more than four half lives of 11C decay. One hour after i.v. administration of 37 MBq 18F-FDG, PET images were acquired over 7 min. List-mode data were reconstructed using ordered-subset expectation maximization with 16 subsets and 4 iterations. The cardiac 18F-FDG uptake was visualized as the percentage of the injected dose per tissue cubic ml (%ID/ml) and an imaging-processing application (AMIDE-bin 1.0.2) was used^34^.

### Effect of specific activity on myocardial 11C-HED uptake

11C-HED was synthesized by N-methylation of (−)-metaraminol as the free base (radiochemical purity >95%) and a wide range of specific activities (0.2–141.9 GBq/μmol) were prepared. Under isoflurane anesthesia, 11C-HED (48.7 ± 9.7 MBq, ranged 0.2–60.4 μg/kg cold mass) was injected via the tail vein in 14 healthy female Wistar rats (Charles River Laboratories, 350–440 g) and dynamic PET images were obtained over 30 min. All animals received approximately the same mass dose of metaraminol (1.5 ± 1.4 μg/kg), the precursor of 11C-HED.

Regions of Interest (ROI) were drawn at the mid-ventricular level in all 14 animals for assessment of uptake in myocardial tissue and at the left atrial cavity* for obtaining blood activity. Cardiac 11C-HED retention index (%/min) was calculated as myocardial tissue activity at 20–30 min divided by the integral of the blood activity curves. The washout rate (%/min) was calculated as follows: (mean cardiac counts_7.5min_ − mean cardiac counts_27.5min_)/(mean cardiac counts_7.5min_) × 100/20 (min). The effect of cold mass on the retention index was evaluated by fitting the data to a dose-response model with variable slope (Equation 1):

1 $$Y={\rm{Bottom}}+({\rm{Top}} \mbox{-} {\rm{Bottom}})/(1+10^{\wedge}(({\rm{LogEC}}50 \mbox{-} {\rm{X}}{)}^{\ast }{\rm{HillSlope}})),$$

where *Y* is the Retention index, *X* is the log of dose, and EC50 is the median effective concentration. Bottom value was estimated from the blood activity at 20–30 min.

The relationship between cold mass and washout rate was assessed by fitting the data to a Michaelis-Menten kinetics (Equation 2):

2 $$Y=({{\rm{rate}}}_{{\rm{\max }}}{\rm{\times }}X)/({{\rm{K}}}_{{\rm{dis}}}+X),$$

where *Y* is the washout rate, *X* is the dose, rate_max_ is the maximum rate of radioactivity loss, and K_dis_ is the half-saturation dose.

### Impact of ageing on myocardial 11C-HED uptake

Serial 11C-HED PET imaging was conducted in 7 healthy male Wistar Rats (Charles River Laboratories) at different ages (month (M) 2, 5, 11 and 15). 11C-HED was synthesized as previously described^35^. Obtained specific radioactivity was 370–740 GBq/μmol and radiochemical purity was >95%. 18F-FDG was synthesized in an in-house cyclotron according to the manufacturer’s instructions.

### Statistical Analysis

All results are displayed as mean ± standard deviation. Statistical analysis was performed using StatMate III (ATMS Co., Ltd., Tokyo, Japan). Statistical significance between the groups was determined by one-way ANOVA followed by post hoc Tukey multiple comparison analysis. A P-value of less than *0*.*05* was assumed to be statistically significant.

## Results

### *In-vivo* blocking study

**Figure 1:**
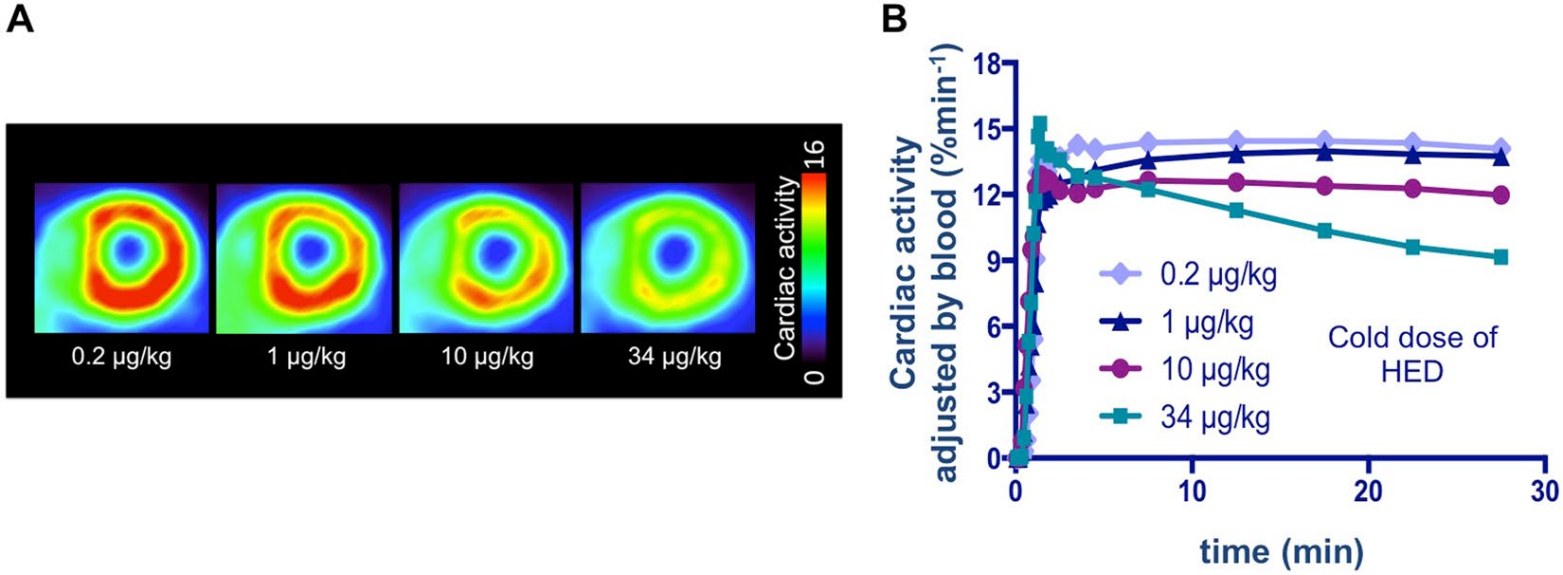
Uptake of radioactivity in the rat heart of 4 different animals (at an age of 2 months) after injection of 11C-HED with different specific activities. (**A**) Representative axial PET images 25–30 min post-injection of 11C-HED. (**B**) Time-activity curves for the myocardium with different amount of cold doses. A dose-dependent reduction of cardiac 11C-HED uptake can be observed.

11C-HED dynamic PET with different tracer specific activities showed rapid blood clearance and clear delineation of the myocardium in all animals. A dose-dependent reduction of cardiac 11C-HED uptake with different specific activities was observed (Fig. 1A, representative axial PET images): With a low dose of 0.2 μg/kg, uptake in the left ventricular myocardium could be clearly visualized, while a slight decrease could be observed at a dose of 1 μg/kg. A further notable decline could be observed with 10 μg/kg, while myocardial uptake almost vanished with 34 μg/kg. Time-activity curves presented in Fig. 1B demonstrated that with the highest cold dose (34 μg/kg), the washout considerably increased, while both the low-dose time-activity curves (0.2 μg/kg and 1 μg/kg) remained stable. The middle-dose time-activity curve (10 μg/kg) demonstrated a moderate increase in washout.

**Figure 2:**
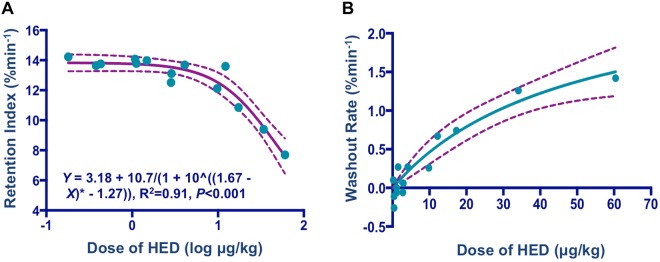
Dose-effect relationships for myocardial uptake of 11C-HED. (**A**) Retention index. The estimated retention indices as a marker of norepinephrine re-uptake function decreased at lower specific activity (i.e. higher amount of cold mass). (**B**) Washout Rate. The washout rate increased as loaded cold mass increased. Every dot represents one investigated animal, dotted lines show 95% inclusion limits and solid lines indicate dose-effect curves.

The estimated retention index as a marker of norepinephrine re-uptake function decreased with lower specific activity (higher amount of cold mass, Fig. 2A). The data were well fitted by a dose–response model (R^2^ = 0.91, P < 0.001) and the EC50 value (95% confidence intervals) was 46.3 (34.6–62) μg/kg. Notably, at a dose of 1 log μg/kg, the retention index decreased at lower specific activity (Fig. 2A). Similar findings were also observed for the washout rate: at a cold dose of 10 μg/kg HED, the washout rate increased markedly as loaded cold mass increased (Fig. 2B). The parameters calculated were rate_max_ = 2.71% min^−1^ and K_dis_ = 48.7 μg/kg.

### Longitudinal 11C-HED imaging

**Figure 3:**
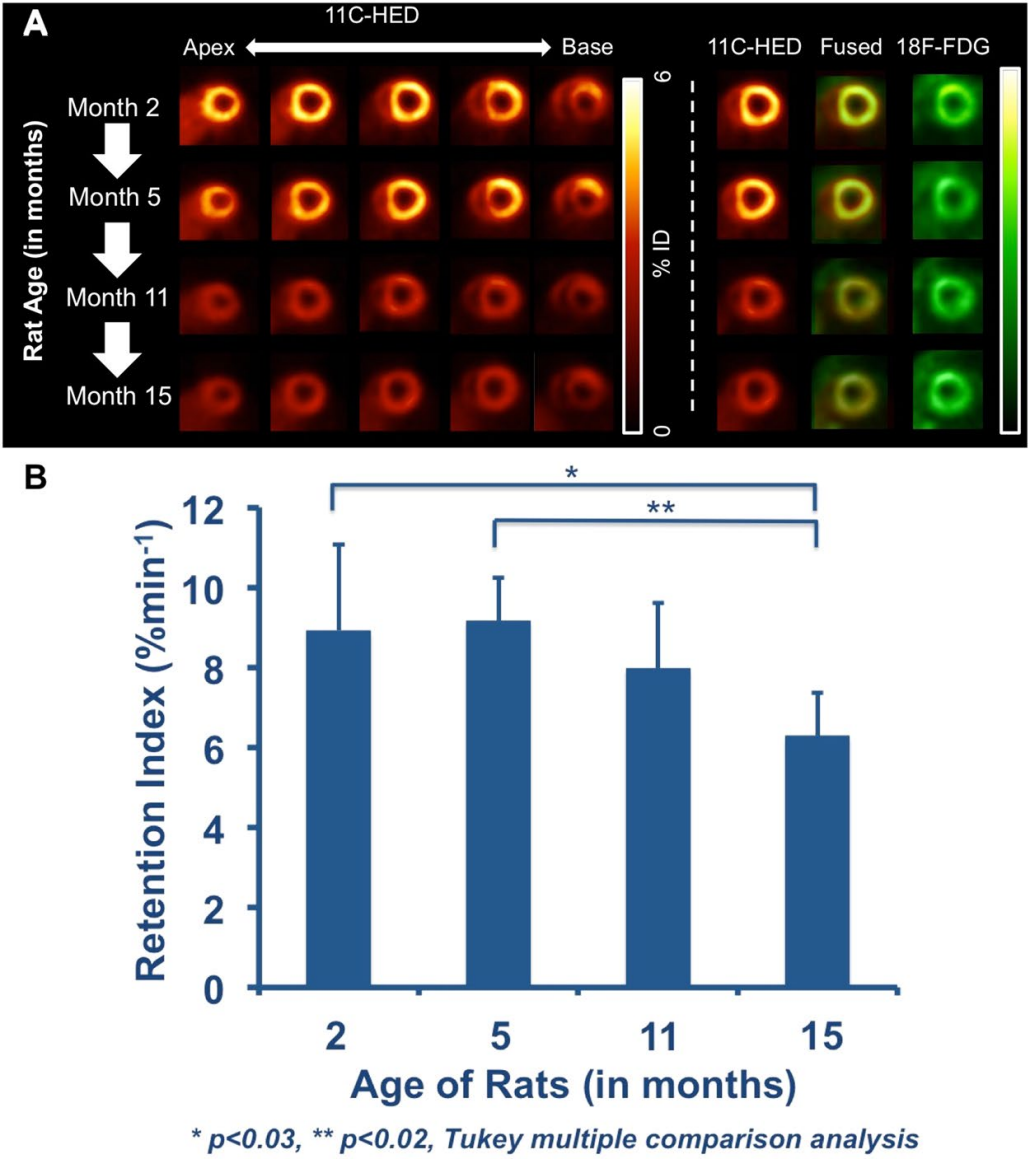
(**A**) *In-vivo* serial PET imaging with 11C-HED and 18F-FDG in healthy Wistar Rats at different ages (Month 2, 5, 11 and 15). An age-related decline of 11C-HED uptake can be observed, whereas 18F-FDG uptake remained stable at different ages. (**B**) Retention indices of rats at different ages (Month 2, 5, 11 and 15). Retention Indices decreased with increasing age (Month 2 and Month 5 vs. Month 15, p < 0.03 and p < 0.02, respectively).

In a longitudinal setting, serial 11C-HED imaging was conducted at different ages of Wistar Rats. 11C-HED PET images demonstrated clear visualization of the left ventricular wall indicating high and homogeneous tracer activity throughout the left ventricular myocardium for all animals at M2. However, the homogenous cardiac uptake pattern reduced subsequently from M5 to M11 and a further decline could be detected at M15. 18F-FDG uptake remained stable throughout the ventricle at different ages, indicating preserved myocardial viability (Fig. 3A). 11C-HED retention indices (%/min) decreased with increasing age (M2: 8.9 ± 2.2, M5: 9.2 ± 1.09, M11: 8 ± 1.64, M15: 6.3 ± 1.1; M2 vs. M15, p < 0.03 and M5 vs. M15, p < 0.02, Fig. 3B).

## Discussion

Cardiac sympathetic nerve PET tracers such as 11C-HED rely on the “uptake-1” recycling pathway^18,19,36^ and the present study demonstrated a high affinity of 11C-HED to the neuronal NE transporter: The estimated retention index as a marker of NE re-uptake function decreased at lower specific activity (i.e. higher amount of cold mass), while the washout rate increased. This observed effect triggered a subsequent investigation: The impact of ageing on 11C-HED uptake was investigated by a longitudinal imaging study in healthy rats and an age-related decline of cardiac sympathetic innervation could be demonstrated.

The high affinity of 11C-HED for neuronal uptake-1 has also been proven previously. However, species- and tracer-dependent variations have to be considered^36,37^: In an *in-vivo* rabbit study, cardiac washout was enhanced by a desipramine chase protocol (i.e. addition of the uptake-1 blocker desipramine immediately after initial tracer accumulation), which suggests a continuous cyclical release (diffusion out) and reuptake of 11C-HED via neural NE transporter in the rabbit heart^33^. Rischpler *et al*. investigated the same species like in the present study (Wistar Rats) and also demonstrated that desipramine led to a reduction of 11C-HED accumulation in the rat myocardium (while 123I-mIBG showed a high contribution to non-neuronal uptake-2)^37^. DeGrado *et al*. further corroborated these observations by using 11C-HED in isolated perfused rat hearts^38^. Hence, given the high affinity of 11C-HED to the neuronal NE transporter as demonstrated in the present study, the rat heart seems to serve as a suitable platform for 11C-HED sympathetic nerve imaging.

Considering the rapid radioactive decay of 11C labeled compounds (20.4 min) compared to 18F (110 min), lower specific activity (higher amount of cold mass) might hamper the diagnostic accuracy of 11C-HED studies. However, this problem can be neglected in a clinical setting, as normally one 11C-HED radiosynthesis is scheduled for one single patient injection. Nevertheless, the herein presented cold mass effect might be of utmost importance in planning small animal PET studies using 11C-HED^39,40^: Analogous to patient care, only one radiotracer production should be considered for one animal, otherwise the cold mass might increase dramatically for the second, succeeding PET study. However, scanning one single animal per radiosynthesis might also lead to a significant cost expansion. Consequently, the findings of the present study should be at least taken into account if one 11C-HED radiosynthesis is scheduled for multiple animals. At a dose of 1 log μg/kg, the retention index decreased at lower specific activity (Fig. 2A). In a similar vein, at a cold dose of 10 μg/kg, the washout rate increased markedly as loaded cold mass increased (Fig. 2B). Thus, those upper limits may serve as practical recommendations for conducting micro PET studies with 11C-HED.

An extensive body of evidence has reported on the general age-related changes in autonomic nervous system function^41,42^. In particular, age-dependent alterations in myocardial sympathetic innervation have attracted interest because of the potential association between cardiac neurohumoral impairment and age-related increase in cardiovascular diseases^4–7,43^. Apart from that, a more frequent use of the cardiac sympathetic nerve tracer 11C-HED can be envisaged in the near future, mainly due to its favourable properties for risk stratification among severe HF patients^21,25^. Therefore, we also aimed to explore the impact of ageing on cardiac 11C-HED uptake in healthy Wistar rats and a decline of myocardial innervation with increasing age could be observed (Fig. 3). However, the concept of assessing an age-related effect on cardiac uptake-1 with neurohumoral PET or SPECT probes is not entirely novel: Tsuchimochi and coworkers demonstrated a decreasing inferior wall uptake in elder, healthy men using 123I-mIBG^27^. In patients suffering from systolic HF, a paralleled decrease in both early and late heart-to-mediastinum ratios with increasing age has been reported^30^. Li *et al*. investigated the F18-labelled neuronal imaging agent fluorodopamine in healthy volunteers and not surprisingly, an uptake reduction in the myocardium along with physiological human ageing could be observed^44^. However, the investigation of a potential age-related impact among different cardiac sympathetic nerve tracers is of utmost importance, as all of these investigated PET or SPECT probes significantly differ in their kinetic properties (e.g., 11C-HED is resistant to degrading enzymes^36^, whereas 6–18F-fluorodopamine shares similar metabolic pathways to physiological NE^44^).

Of note, Bernacki *et al*. recently compared a younger patient cohort (18–33 y) vs. an older cohort (65–80 y) and reported on a decline in cardiac sympathetic nerve function assessed by 11C-HED PET. Although extrapolations from preclinical observations to humans must be done with extreme caution, the herein presented age-related decrease of sympathetic nerve function in the rat myocardium corroborates these previously reported findings^45^. However, due to the preclinical setting of the present study, the same rat could be imaged at different time points of its life, which might be comparable to different stages in a human life cycle^46^: M2 in a rat life corresponds approximately to early/middle childhood (human age, 6 y), M5 to adolescence (12–20 y), M11 to early adulthood/ midlife (35–50 y) and M15 to mature adulthood (50–70 y). Hence, in contrast to Bernacki *et al*. selecting two extremes (adolescence vs. late adulthood)^45^, the present study not only reports on significant differences in cardiac nerve function between young and old (M2 vs. M15 group), but also on an age-dependent loss of myocardial innervation over the life span of a healthy rat.

## Conclusions

In a longitudinal 11C-HED imaging study in healthy rats, an age-related decline on myocardial sympathetic nerve activity could be demonstrated, which is consistent with the generalized decrease of peripheral somatic nerve function in the elderly. However, the herein reported cold mass effect might be of utmost importance in planning micro PET studies: only one production should be considered for one animal, as the cold mass might increase in succeeding scans for multiple animals.
